# Aerosolization Performance of Immunoglobulin G by Jet and Mesh Nebulizers

**DOI:** 10.1208/s12249-023-02579-8

**Published:** 2023-05-24

**Authors:** Kyung Hwa Chang, Bong Joo Park, Ki Chang Nam

**Affiliations:** 1 Department of Medical Engineering, Dongguk University College of Medicine, 32 Dongguk-ro, Ilsandong-gu, Goyang-si, Gyeonggi-do 10326 South Korea; 2 Department of Electrical & Biological Physics and Institute of Biomaterials, Kwangwoon University, Seoul, 01897 South Korea

**Keywords:** antibody, drug delivery, immunoglobulin G, nebulizer, respiratory therapy

## Abstract

Recently, many preclinical and clinical studies have been conducted on the delivery of therapeutic antibodies to the lungs using nebulizers, but standard treatment guidelines have not yet been established. Our objective was to compare nebulization performance according to the low temperature and concentration of immunoglobulin G (IgG) solutions in different types of nebulizers, and to evaluate the stability of IgG aerosols and the amount delivered to the lungs. The output rate of the mesh nebulizers decreased according to the low temperature and high concentration of IgG solution, whereas the jet nebulizer was unaffected by the temperature and concentration of IgG. An impedance change of the piezoelectric vibrating element in the mesh nebulizers was observed because of the lower temperature and higher viscosity of IgG solution. This affected the resonance frequency of the piezoelectric element and lowered the output rate of the mesh nebulizers. Aggregation assays using a fluorescent probe revealed aggregates in IgG aerosols from all nebulizers. The delivered dose of IgG to the lungs in mice was highest at 95 ng/mL in the jet nebulizer with the smallest droplet size. Evaluation of the performance of IgG solution delivered to the lungs by three types of nebulizers could provide valuable parameter information for determination on dose of therapeutic antibody by nebulizers.

**Graphical abstract:**

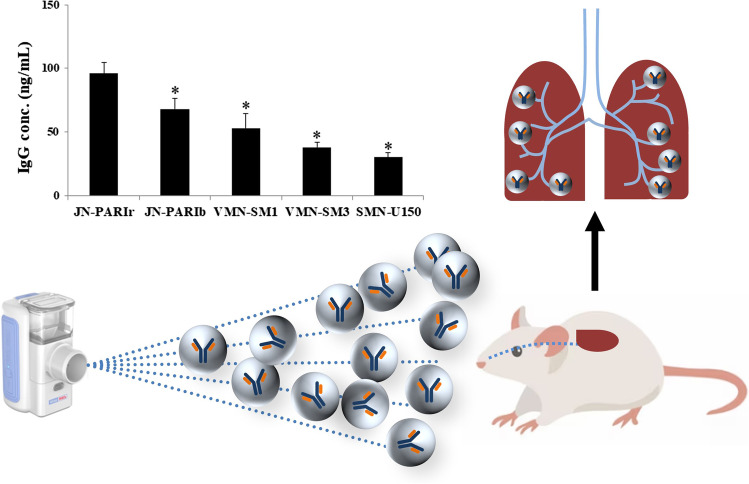

## Introduction

Several therapeutic monoclonal antibodies have recently been approved or are currently under clinical evaluation for the treatment of respiratory diseases in pulmonary delivery of antibodies [1, 2]. In particular, interest is increasing as antibodies can be used to treat pneumonia patients due to multidrug-resistant bacteria or emergent viruses before vaccines for respiratory infectious diseases such as SARS-CoV2 and MERS-CoV are developed [3, 4]. The advantage of using antibodies for treatment is that they have excellent target specificity, high target binding affinity, and a desirable safety profile compared to small-molecule drugs [5]. Current antibody therapeutics is limited to needle-based injection delivery routes to secure high bioavailability because of poor stability through oral administration [6]. However, recent reports have shown that antibodies developed for intravenous delivery are often quite limited in the amounts of antibodies delivered to the lung tissue following systemic delivery [7]. Pulmonary delivery to target lung sites (lower airways and alveoli) has the potential benefit of increasing limited drug concentrations to the lungs with small doses of the drug, reducing toxicity and side effects from intravenous or oral administration [8, 9].

The development of inhaled drugs for the treatment of respiratory diseases in the past decades has mainly focused on small molecules. To date, only one inhaled protein for the treatment of cystic fibrosis has been approved by the FDA [2, 10]. Unlike approved small molecules, Pulmozyme® prescribing information recommends only five jet nebulizers and one vibrating mesh nebulizer that have completed preclinical and clinical research on aerosolization performance, aerosol stability, delivery efficiency, safety, dose, efficacy, and adverse effects [11–13]. Most of the approved therapeutic monoclonal antibodies belong to the immunoglobulin G1 subclass, which are significantly larger than small molecules and have complex physicochemical properties [5]. The relationship between the operating principle of nebulizers and the physicochemical properties of antibody has effect on the stability of aerosolized antibodies for pulmonary delivery [14]. During aerosolization with a nebulizer, proteins are exposed to various stresses, such as high temperature, ultrasonic, cavitation, and shear. In response to these stresses, proteins tend to undergo inactivation of their biological activities due to unfolding and aggregation and induce unexpected immune responses, thus resulting in pharmacological and safety issues. Aggregation can generally be considered a standard marker of instability for aerosol of protein [15, 16]. Therefore, there are many challenges to be overcome during the development of inhaled antibodies, and the advantages of the inhalation route for therapeutic antibodies have not yet come to fruition in clinical practice.

There are three different types of nebulizers: jet, ultrasonic, and mesh nebulizers, depending on their operating principles. Jet nebulizer has been used for a long time in the treatment of pulmonary disease, but it is non-portable because of its weight and vibration by air compressor [17]. Ultrasonic nebulizer has also limitations, such as thermal and mechanical stresses, making it difficult to use them for heat sensitive proteins [17]. Mesh nebulizer uses piezoelectric elements (lead zirconate titanate, PZT) and mesh filters to improve aerosol generation efficiency with less residual volume and high patient convenience [18]. A mesh is attached to ring shape of PZT in vibrating mesh nebulizer, while static mesh nebulizer use an ultrasonic horn to vibrate liquid drug and push aerosols towards the static mesh [19].

Among the various types of nebulizers, mesh nebulizer has been mainly used proteins for inhalation [8, 16, 20]. According to Respaud *et al.* and Bodier-Montagutelli *et al.*, it was reported that mesh nebulizer is suitable for efficient delivery of high doses of protein into the lungs [8, 16]. In general, most small molecule inhalants for respiratory diseases are stored at room temperature and can be used immediately after storage in various types of nebulizers. Therapeutic antibodies, on the other hand, are temperature-sensitive proteins that are stored under cold-chain conditions to maintain stability and biological activity [21]. Studies on the nebulization of therapeutic antibodies using nebulizers have been conducted without considering the temperature of the antibody solution in the different types of nebulizers. In addition, high viscosity of therapeutic antibodies is known to be a factor that can significantly affect stability and drug delivery efficiency [22]. The nebulization performance may be affected when a cold antibody solution is immediately applied to the nebulizer because commercial nebulizers have different operating principles.

In this study, we evaluated the nebulization performance according to the temperature and physicochemical characteristics of immunoglobulin G (IgG) solutions in the different types of nebulizers. Protein integrity was evaluated between nebulized and non-nebulized solution using electrophoresis with two different polyacrylamide gels and aggregation assay by fluorescent dye. We also compared the amount of IgG delivered to the lungs of mice treated with different types of nebulizers.

## Materials and Methods

### Materials and Nebulizers

Intravenous human IgG solution (10%, 100 mg/mL) was acquired from GC Pharma (formerly Green Cross Corporation, Yongin, Korea). In the subsequent nebulization experiments, IgG was diluted with saline to 1, 10, 20, and 40 mg/mL. A DC protein assay kit and 4–15% Mini-PROTEAN® TGX™ precast Protein Gels were purchased from Bio-Rad Laboratories (Hercules, CA, USA). PROTEOSTAT® Protein aggregation assay and IgG Human ELISA Kit were purchased from Enzo Life Sciences (Farmingdale, NY, USA) and Thermo Fisher Scientific (Waltham, MA, USA), respectively. Five nebulizers, with three different operating types, were used in this study (Table I). The PARI BOY SX + LC SPRINT was used with red and blue nozzles. PZT the same used in VMN-SM1 and VMN-SM3 was provided by KTMED (Seoul, Korea).

**Table I:** Nebulizers Used in the Study

Mode of nebulizer	Model/manufacture	Abbreviation in the study
Jet	PARI BOY SX + LC SPRINT with red nozzle/PARI GmbH, Starnberg, Germany	JN-PARIr
	PARI BOY SX + LC SPRINT with blue nozzle/PARI GmbH, Starnberg, Germany	JN-PARIb
Vibrating mesh	NE-SM1 NEPLUS/KTMED Co., Seoul, Korea	VMN-SM1
	NE-SM3/KTMED Co., Seoul, Korea	VMN-SM3
Static mesh	NE-U150/Omron Healthcare, Kyoto, Japan	SMN-U150

### Animals

Animal experiments were approved by the Ethics Committee of the Animal Service Center at Dongguk University (IACUC Number:2021-021-2, September 02, 2023). Eight-week-old female BALB/c mice were purchased from Daehan Biolink (Seoul, Korea).

### Nebulization Performance of IgG

Saline was stored in a refrigerator or at RT (20–22°C), and IgG (1, 10, 20 and 40 mg/mL) solutions were stored in a refrigerator until use. Cold saline (Cold_S), RT saline (RT_S), and cold IgG (Cold_IgG) stored refrigerated or at RT were used immediately for nebulization. The storage of IgG at RT was not considered because it did not comply with the storage instructions. RTequ_S (RT equilibrated) and RTequ_IgG (RT equilibrated IgG) were the saline and IgG that were taken out of refrigerator allowed to equilibrate its temperature at RT for 15 min prior to being placed in a reservoir of nebulizer. After filling the reservoir with 2 mL of the analytical solution, jet nebulizers were continuously operated until 1 min after the beginning of sputtering. The top, insert, and cup of jet nebulizer were weighed before and after nebulization, and mesh nebulizer weights were measured at 1-min intervals on an analytical balance. The mesh nebulizers were operated until the aerosol generation of the saline stopped or IgG output rate decreased suddenly. The nebulization times were recorded using a timer, and the residual volume was determined gravimetrically [23]. The output rate of nebulizer was calculated using the following equation:

$$Output\ Rate\ \left(\frac{mL}{\mathit{\min}}\right)=\frac{charged\ volume\ (mL)- residual\ volume\ (mL)}{nebulization\ time\ \left(\mathit{\min}\right)}$$

### Viscosity Measurement of IgG

Viscosity measurements of IgG (1, 10, 20, and 40 mg/mL) were conducted by KBIO Health (Osong Medical Innovation Foundation, Korea). A m-VROC™ viscometer (RheoSense Inc., CA, USA) was operated at 25°C to measure the viscosity of each IgG solution. The IgG solution was loaded as approximately 0.25 mL using a syringe.

### Electrical Impedance Measurements of PZT

Saline and IgG (1, 10, 20, and 40 mg/mL) solutions were stored in a refrigerator until use, and RT-equilibrated saline (RTequ_S) and RT-equilibrated IgG (RTequ_IgG) were allowed to stand for 15 min at RT prior to impedance measurement. An impedance analyzer (E4980A, Keysight, CA, USA) was used to measure the impedance of the PZT. The impedance was measured at 108 kHz, which is the nominal frequency used by the manufacturer to drive the PZT.

### Droplet Size Assessment of IgG Aerosol

The distribution of IgG aerosol size (mass median diameter, MMD) was measured by the laser diffraction method using a Spraytec System (Model #STP5311, Malvern instrument, Malvern, UK) [24]. Saline and IgG (1, 10, 20, and 40 mg/mL) were allowed to equilibrate at RT for 15 min prior to nebulization. The volume median diameter (Dv50) of aerosol was calculated using a Spraytec software version 3.1 (Malvern Instruments).

### IgG Quantification in the Residual Volume

To quantify IgG concentration in the residual volume after IgG nebulization, control samples (non-nebulized IgG; nominal IgG) were kept at RT until the nebulization was complete for each IgG solution. IgG concentration for each residual volume was measured using a DC protein assay kit according to the manufacturer’s protocol. In the case of a high-concentration IgG sample, it was measured after dilution with saline. The absorbance was measured at 750 nm using a Spark® 10M multimode reader (Tecan, Zurich, Switzerland). The concentration of nominal IgG was taken as 100% and the relative concentration of IgG was calculated.

### Temperature Monitoring During Nebulization

The temperature of the IgG in the reservoir during nebulization was recorded at 1-min interval using a digital multimeter with a K-type thermocouple probe (FLUKE-87-5, Fluke, Everett, WA, USA) [25]. The thermocouple is in contact with the solution inside the reservoir. The saline and IgG solutions were stored in a refrigerator until use and allowed to equilibrate to RT for 15 min immediately prior to use. Cold_S and Cold_IgG conditions were excluded because the temperature problem focused on the RT equilibrium condition.

### Polyacrylamide Gel Electrophoresis (PAGE) for Aerosolized IgG

To collect IgG aerosols, 50-mL disposable plastic centrifuge tubes and mouthpieces were connected to each nebulizer. The collected IgG was immediately centrifuged at 2500 × g for 1 min and transferred to clean microtube and stored in a refrigerator until required for analysis. Control samples (nominal IgG) were stored at RT, while each IgG solution was nebulized and then stored in a refrigerator. Following protein quantification for the collected IgG samples, IgG samples (15 μg/20 μL) were mixed with 5X SDS-PAGE sample loading buffer. After electrophoresis using 4–15% Mini-PROTEAN® TGX™ Precast Gels (native polyacrylamide gels) or 15% SDS polyacrylamide gels, the gels were stained [25].

### Protein Aggregation Assay for Aerosolized IgG

The aggregation of IgG aerosols was determined using a PROTEOSTAT^®^ Protein aggregation assay. Collected IgG samples and control samples (nominal IgG) were kept on ice for approximately 1 h during determination of IgG concentration and preparation of diluted IgG aerosol (100 μg/100 μL) samples. The diluted IgG aerosol samples were transferred to a well containing 2 μL of PROTEOSTAT® detection reagent. Control samples were heated for 5 min and used as a positive control (PC). The well plate was incubated for 15 min at RT and the fluorescence intensity was then measured with excitation at 480 nm and emission at 600 nm using a Spark™ 10M. The relative aggregation of IgG aerosol samples was calculated using the fluorescence intensity of the heated control sample (PC) as 100%.

### Exposure of Mice to IgG and Quantification of Delivered IgG

The closed head-only exposure chamber specifically designed in our previous study was used to expose anesthetized mice to 2 mg IgG (1 mg/mL) [25], and nine mice were used for each group. The bronchoalveolar lavage fluid (BALF) samples were collected by typical procedure with 0.8 mL warm saline immediately after completion of IgG exposure before euthanasia. BALF samples were centrifuged, and supernatants were transferred to pre-cooled tubes and immediately frozen at −80°C. Quantification of IgG in BALF was assessed by a human IgG ELISA Kit. The BALF samples were diluted 2–5 times with saline before being assayed and then the optical density was measured at 450 nm using a Spark™ 10M.

### Statistical Analysis

Data were expressed as mean and standard deviation (SD). One-way or repeated two-way analysis of variance followed by Dunnett’s test was performed using SigmaPlot software version 13 (Systat Software, San Jose, CA, USA). A *p*-value less than 0.05 was considered statistically significant.

## Results

### *In Vitro* Nebulization Performance for Cold and RT Solutions

The residual volume, nebulization time, and output rate were evaluated to compare the performance of the nebulizers according to the temperature of the solution for both RT saline (RT_S) and cold saline (Cold_S). Table II shows the well-known characteristics of mesh nebulizers with small residual volumes and short nebulization times, whereas jet nebulizers have incomplete nebulization resulting in their large residual volumes in both RT_S and Cold_S [17, 26]. JN-PARIr had the longest nebulization time among the five nebulizers, owing to its lowest output rate. The jet nebulizers had no significant difference in the residual volume, output rate, and nebulization time between RT_S and Cold_S. The residual volumes of RT_S *versus* Cold_S in the three mesh nebulizers were also similar, but the output rate was significantly decreased, and the nebulization time was also significantly increased. The increase in nebulization time required to complete the nebulization of Cold_S in VMN-SM1, VMN-SM3, and SMN-U150 was caused by a decrease in the output rate.

**Table II:** Residual Volumes, Output rates, and Nebulization Times for Saline with Different Temperature in Five Nebulizers

Device	Residual volume (mL)		Nebulization time (min)		Output rate (mL/min)	
	RT_S	Cold_S	RT_S	Cold_S	RT_S	Cold_S
JN-PARIr	0.621 ± 0.019	0.636 ± 0.014	9.61 ± 0.167	9.58 ± 0.083	0.143 ± 0.002	0.142 ± 0.001
JN-PARIb	0.758± 0.014	0.751 ± 0.016	6.97 ± 0.048	7.06 ± 0.096	0.178 ± 0.002	0.177 ± 0.002
VMN-SM1	0.036 ± 0.003	0.031 ± 0.015	6.30 ± 0.048	7.08 ± 0.083^*^	0.311 ± 0.002	0.278 ± 0.005^*^
VMN-SM3	0.020 ± 0.008	0.027 ± 0.005	4.31 ± 0.048	5.00 ± 0.167^*^	0.460 ± 0.007	0.395 ± 0.014^*^
SMN-U150	0.381 ± 0.005	0.365 ± 0.022	3.55 ± 0.164	4.50 ± 0.088^*^	0.457 ± 0.020	0.363 ± 0.012^*^

All data are presented as mean ± SD value. *p < 0.05 *versus* RT_S

**Fig. 1:**
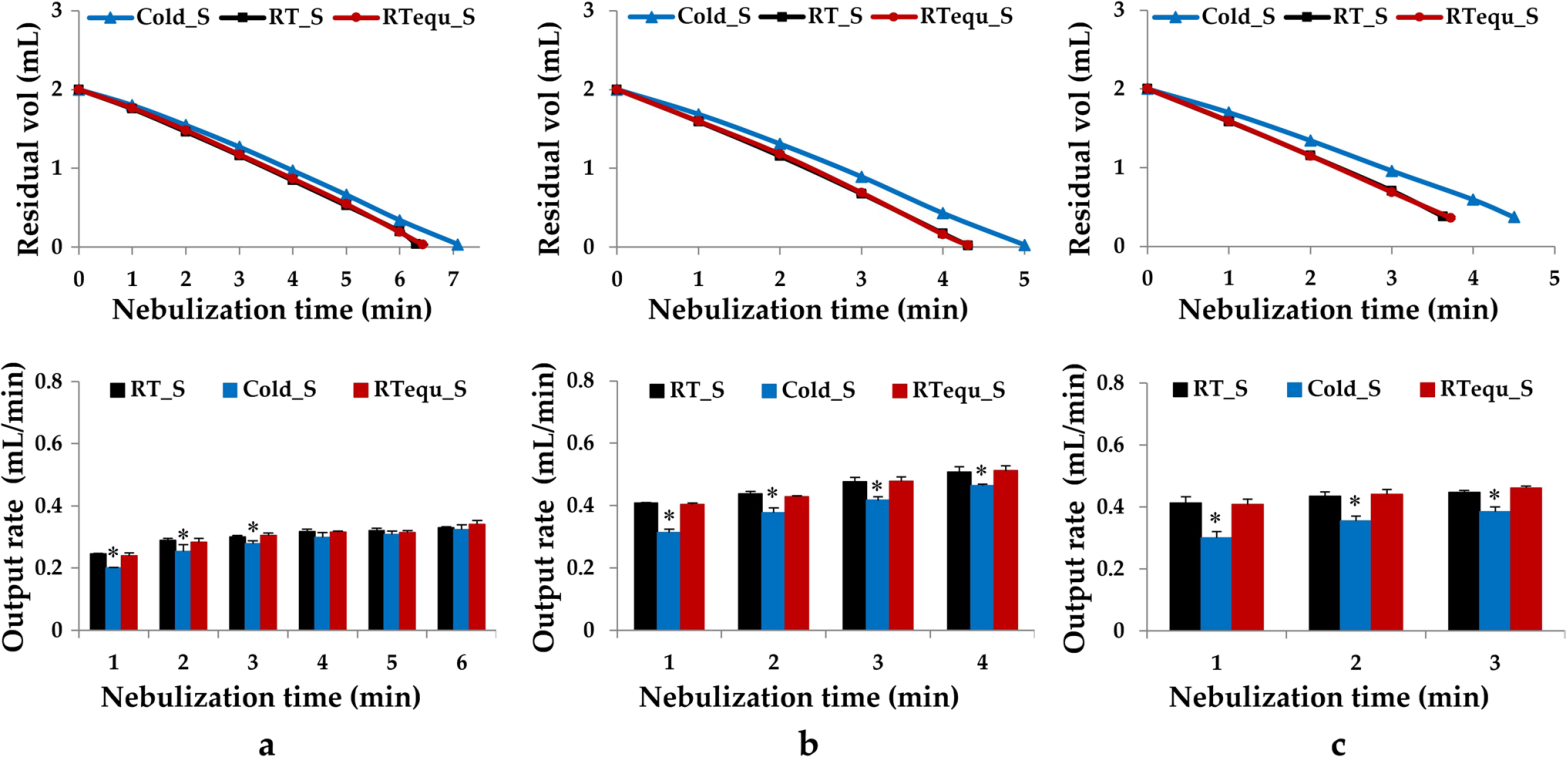
Change in output rates and residual volumes during nebulization with RT saline (RT_S: black bars and square data points), cold saline (Cold_S: blue bars and triangular data points), and cold saline equilibrated at RT for 15 min (RTequ_S: red bars and circular data points) using three mesh nebulizers, VMN-SM1 (**a**), VMN-SM3 (**b**), and SMN-U150 (**c**). All data are presented as mean ± SD value. Asterisk (*) *p* < 0.05 *versus* RT_S

The nebulization performance of cold saline equilibrated at RT for 15 min (RTequ_S) was evaluated to confirm whether the performance degradation due to the effects of low-temperature could be reversed. Figure 1 shows the nebulization time and output rate at 1-min intervals for the three mesh nebulizers for RT_S, Cold_S, and RTequ_S. The nebulization time of RTequ_S in the mesh nebulizers was similar to that of RT_S because the output rate of RTequ_S recovered as much as RT_S. In VMN-SM1, VMN-SM3, and SMN-U150, the output rate reduction of Cold_S was the highest in the initial first minute of nebulization, 18%, 23%, and 27%, respectively. The difference in the output rate between RT_S and Cold_S gradually recovered over time.

### *In Vitro* Nebulization Performance for IgG Solution

RT equilibration conditions were applied when nebulizing a cold IgG formulation, which was developed for IV injection using five different nebulizers. Table III shows the results of the residual volumes, output rates, and nebulization times between Cold_IgG and RTequ_IgG in both jet nebulizers over IgG concentration. There were no significant differences in residual volumes, output rates, and nebulization times between Cold_IgG and RTequ_IgG, as also the case in the saline group. In addition, no difference was observed in the nebulization performance of the two jet nebulizers according to IgG concentration.

**Table III:** Nebulization Performance for IgG with Different Temperatures and Concentrations Using Two Jet Nebulizers

Device	IgG conc. (mg/mL)	Residual volume (mL)		Nebulization time (min)		Output rate (mL/min)	
		Cold	RTequ	Cold	RTequ	Cold	RTequ
JN-PARIr	Saline	0.635 ± 0.014	0.621 ± 0.019	9.58 ± 0.083	9.61 ± 0.096	0.142 ± 0.001	0.143 ± 0.002
	1	0.618 ± 0.029	0.622 ± 0.008	9.69 ± 0.048	9.80 ± 0.127	0.143 ± 0.003	0.141 ± 0.002
	10	0.631 ± 0.024	0.618 ± 0.025	9.61 ± 0.096	9.55 ± 0.096	0.142 ± 0.003	0.145 ± 0.003
	20	0.647 ± 0.011	0.65 ± 0.030	9.58 ± 0.083	9.72 ± 0.096	0.141 ± 0.001	0.139 ± 0.005
	40	0.638 ± 0.017	0.633 ± 0.039	9.44 ± 0.096	9.50 ± 0.083	0.144 ± 0.003	0.144 ± 0.004
JN-PARIb	Saline	0.751± 0.016	0.758 ± 0.014	7.06 ± 0.096	6.97 ± 0.048	0.177± 0.002	0.178 ± 0.002
	1	0.636± 0.014^*^	0.634 ± 0.018^*^	7.69 ± 0.048^*^	7.56 ± 0.096^*^	0.177 ± 0.001	0.180 ± 0.003
	10	0.672± 0.017^*^	0.653 ± 0.016^*^	7.61 ± 0.096^*^	7.47 ± 0.173^*^	0.174 ± 0.002	0.180 ± 0.007
	20	0.660± 0.025^*^	0.650 ± 0.023^*^	7.64 ± 0.048^*^	7.44 ± 0.096^*^	0.177 ± 0.002	0.181 ± 0.005
	40	0.676± 0.005^*^	0.654 ± 0.018^*^	7.64 ± 0.048^*^	7.53 ± 0.048^*^	0.175 ± 0.002	0.179 ± 0.003

All data are presented as mean ± SD value. *p < 0.05 *versus* saline

**Fig. 2:**
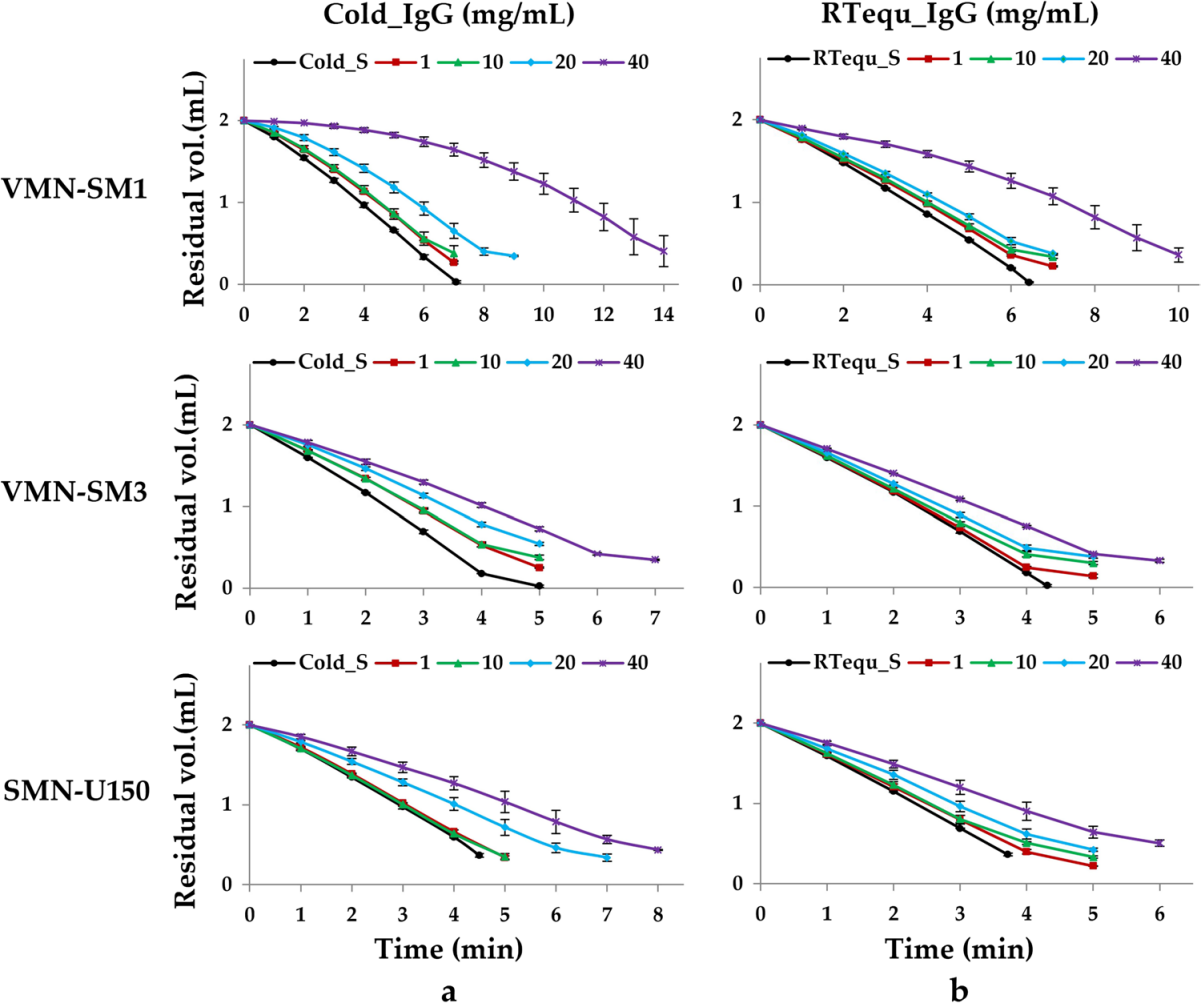
Residual volume and nebulization time change during nebulization on the Cold_IgG (**a**) and RTequ_IgG (**b**) using three mesh nebulizers; VMN-SM1, VMN-SM3, and SMN-U150 (1 mg/mL: red line/square data points, 10 mg/mL: green line/ triangular data points, 20 mg/mL: blue line/diamond-shaped data points, 40 mg/mL: purple line/ cross-shaped data points). Cold_S and RTequ_S were used as controls in cold IgG (**a**; Cold_IgG) and cold IgG equilibrated at RT for 15 min (**b**; RTequ_IgG), respectively. All data are presented as mean ± SD value

**Fig. 3:**
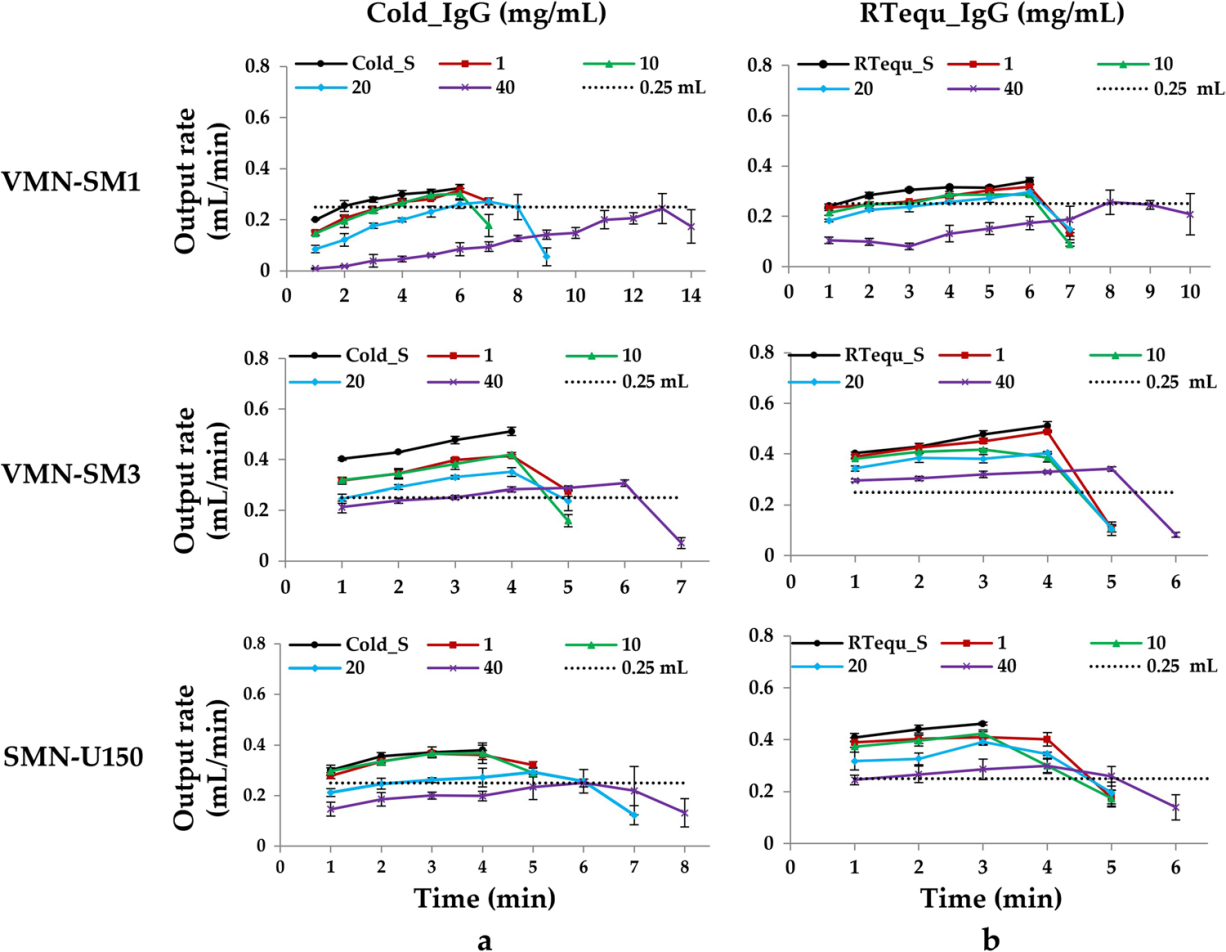
Output rate change during nebulization on the Cold_IgG (**a**) and RTequ_IgG (**b**) using three mesh nebulizers using three mesh nebulizers; VMN-SM1, VMN-SM3, and SMN-U150 (1 mg/mL: red line/square data points, 10 mg/mL: green line/triangular data points, 20 mg/mL: blue line/diamond-shaped data points, 40 mg/mL: purple line/cross-shaped data points). Cold_S and RTequ_S were used as controls in cold IgG (**a**; Cold_IgG) and cold IgG equilibrate at RT for 15 min (**b**; RTequ_IgG), respectively. All data are presented as mean ± SD value

Unlike saline, VMN-SM1 and VMN-SM3 nebulizers showed incomplete nebulization, with large residual volumes in both Cold_IgG and RTequ_IgG due to the generation of numerous foams during nebulization Fig. 2a and b. When foam contacts the mesh during nebulization, the output rate decreased rapidly. The output rate of RTequ_IgG was higher than that of Cold_IgG in all the mesh nebulizers Fig. 3a and b. The output rate decreased with increasing IgG concentrations for both Cold_IgG and RTequ_IgG. VMN-SM1 and SMN-U150 did not meet the manufacturer’s nebulization rate (same as output rate, 0.25 mL/min) specification at higher concentration IgG of Cold_IgG. RTequ_IgG met the manufacturer’s nebulization rate specifications, except for 40 mg/mL VMN-SM1. In particular, 40 mg/mL Cold_IgG of VMN-SM1 was barely nebulized at the beginning of nebulization, but 40 mg/mL RTequ_IgG could be nebulized at an output rate of 0.1 mL/min.

The three mesh nebulizers resulted in longer nebulization times with a reduced output rate, as IgG concentration increased in both Cold_IgG and RTequ_IgG Figs. 2 and 3. However, the nebulization time of RTequ_IgG was shorter than that of Cold_IgG at all IgG concentrations, owing to the increased output rate. VMN-SM1, which had the lower output rate among the mesh nebulizers, greatly reduced the nebulization time from 14 min to 10 min by RT equilibration. Although it is difficult to directly compare the residual volume owing to the generated foam, IgG remaining in the reservoir of RTequ_IgG at the same nebulization time is less than that of Cold_IgG.

### Effects of Low Temperature and the Viscosity of IgG on the Impedance of PZT

**Fig. 4:**
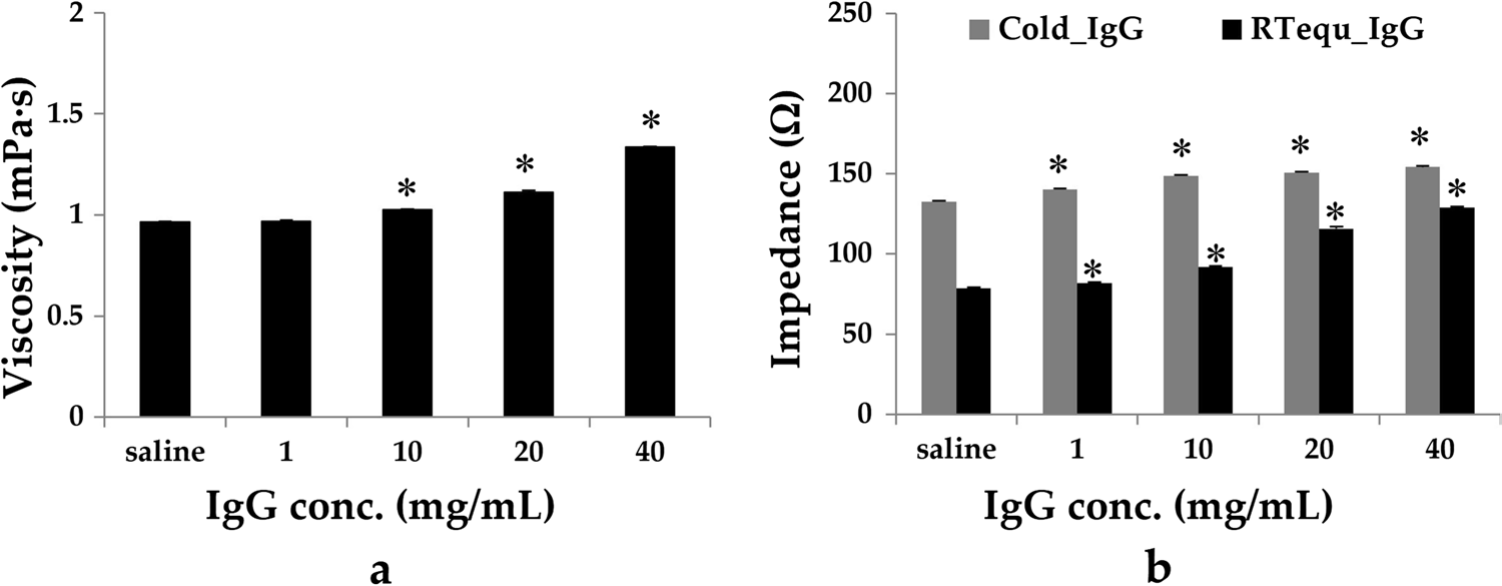
The viscosity (**a**) and PZT impedance (**b**) change depending on the temperature or concentration of IgG solution. Saline was used as control in both assays. All data are presented as mean ± SD value. Asterisk (*) *p* < 0.05 *versus* saline

It has been reported that the viscosity of the antibody solution increases as the concentration increases due to the interaction between several non-shared molecules [27, 28]. As shown in Fig. 4a, the viscosity of IgG solution at RT increased as a function of IgG concentration. The highest viscosity was 1.34 mPa∙s at 40 mg/mL IgG, which is approximately 35% higher than that of saline. This viscosity is similar to that of a 13% glycerol solution [29]. It is known that a change in resonance frequency of the PZT is necessary to adapt to the change in impedance due the temperature of PZT and solution viscosity [30, 31]. Impedance of the PZT increased as IgG concentration increased in both IgG temperature conditions Fig. 4b. The impedance of PZT in Cold_S increased significantly by 70% compared to RTequ_S, and was also higher than the impedance measured at 40 mg/mL RTequ_IgG. Cold_IgG (40 mg/mL) was measured to have the highest impedance owing to its low temperature and high viscosity. Increasing the impedance of PZT by the low temperature and viscosity of the liquid drug can lead to mismatch in the resonant frequency, thereby reducing the output rate of the mesh nebulizer.

### Droplet Sizes of IgG Aerosol and Concentration in Residual Volume

The MMD of the aerosol droplets for saline and RTequ_IgG (1, 10, 20, and 40 mg/mL) emitted by five nebulizers was measured. Table IV shows the differences in IgG aerosol droplet sizes between the five nebulizers. As the Dv50 of each IgG solution was similar to that of saline, there was no difference in the Dv50 according to IgG concentration. JN-PARIr produced the smallest droplets at 3.09 ± 0.04 μm, while VMN-SM3 and SMN-U150 were the largest droplets at 6.70 ± 0.01 μm and 6.66 ± 0.03 μm, respectively.

**Table IV:** Aerosol Droplet Sizes for IgG Solution by Five Nebulizers

Device	IgG of the fifty percent volume diameter (Dv (50) (μm))					
	0 (saline)	1 mg/mL	10 mg/mL	20 mg/mL	40 mg/mL	
JN-PARIr	3.00 ± 0.07	3.21 ± 0.04	3.10 ± 0.07	3.19 ± 0.01	2.95 ± 0.06	
JN-PARIb	5.20 ± 0.01	5.55 ± 0.02	5.59 ± 0.07	5.53 ± 0.03	5.34 ± 0.04	
VMN-SM1	4.58 ± 0.05	4.72 ± 0.05	4.67 ± 0.05	4.75 ± 0.01	4.92 ± 0.03	
VMN-SM3	6.94 ± 0.12	6.75 ± 0.12	6.74 ± 0.19	6.53 ± 0.05	6.52 ± 0.01	
SMN-U150	6.45 ± 0.03	6.69 ± 0.01	6.68 ± 0.01	6.80 ± 0.07	6.69 ± 0.03	

**Fig. 5:**
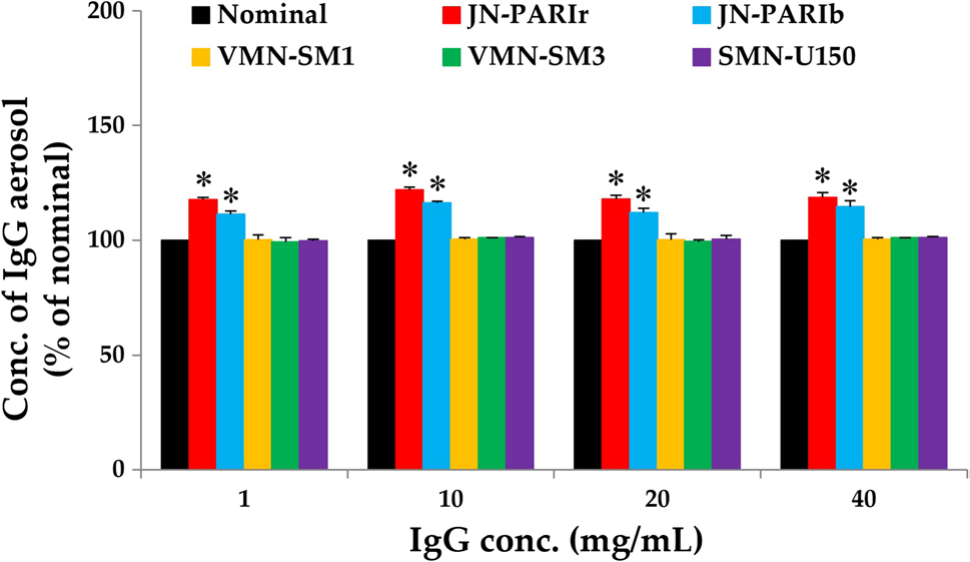
The change IgG concentration in the reservoir after nebulization with IgG solution using five nebulizers; JN-PARIr: red bars, JN-PARIb: blue bars, VMN-SM1: orange bars, VMN-SM3: green bars, and SMN-U150: purple bars. The concentration of non-nebulized nominal IgG was defined as 100% (black bar), and the relative concentration of IgG was calculated. All data are presented as mean ± SD value. Asterisk (*) *p* < 0.05 *versus* nominal samples

IgG concentration in the residual volume after nebulization by the two jet nebulizers increased by approximately 11–21% compared to the non-nebulized nominal samples, whereas there was no change in the three mesh nebulizers Fig. 5. The result for the jet nebulizer was consistent with previous study [17].

### Temperature in the Reservoir During IgG Nebulization

**Fig. 6:**
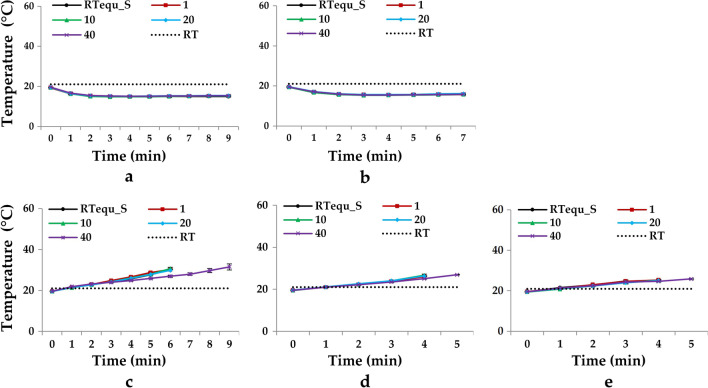
Plot of reservoir temperature changes during nebulization with IgG solution (RTequ_S: black line/circular data points, 1 mg/mL: red line/square data points, 10 mg/mL: green line/triangular data points, 20 mg/mL: blue line/diamond-shaped data points, and 40 mg/mL: purple line cross-shaped data points) by the JN-PARIr (**a**), JN-PARIb (**b**), VMN-SM1 (**c**), VMN-SM3 (**d**), and SMN-U150 (**e**). The black dashed lines indicate RT. All data are presented as mean ± SD value

Temperature of the reservoir during nebulization was investigated because it may affect protein activity, structure, and stability [15]. The temperatures of the RTequ_S and RTequ_IgG in the JN-PARIr and JN-PARIb reservoirs decreased rapidly to 16 ± 1°C below RT within 1 min after operation and were maintained until the nebulization was complete Fig. 6a and b. However, three mesh nebulizers were raised the temperature from 4 to 13°C above the temperature at the baseline in both RTequ_S and RTequ_IgG Fig. 6c, d, and e. VMN-SM1 reached temperatures as high as 34°C because the nebulization time was longer than those of VMN-SM3 and SMN-150.

### Primary Structure Integrity of Aeroslized IgG

**Fig. 7:**
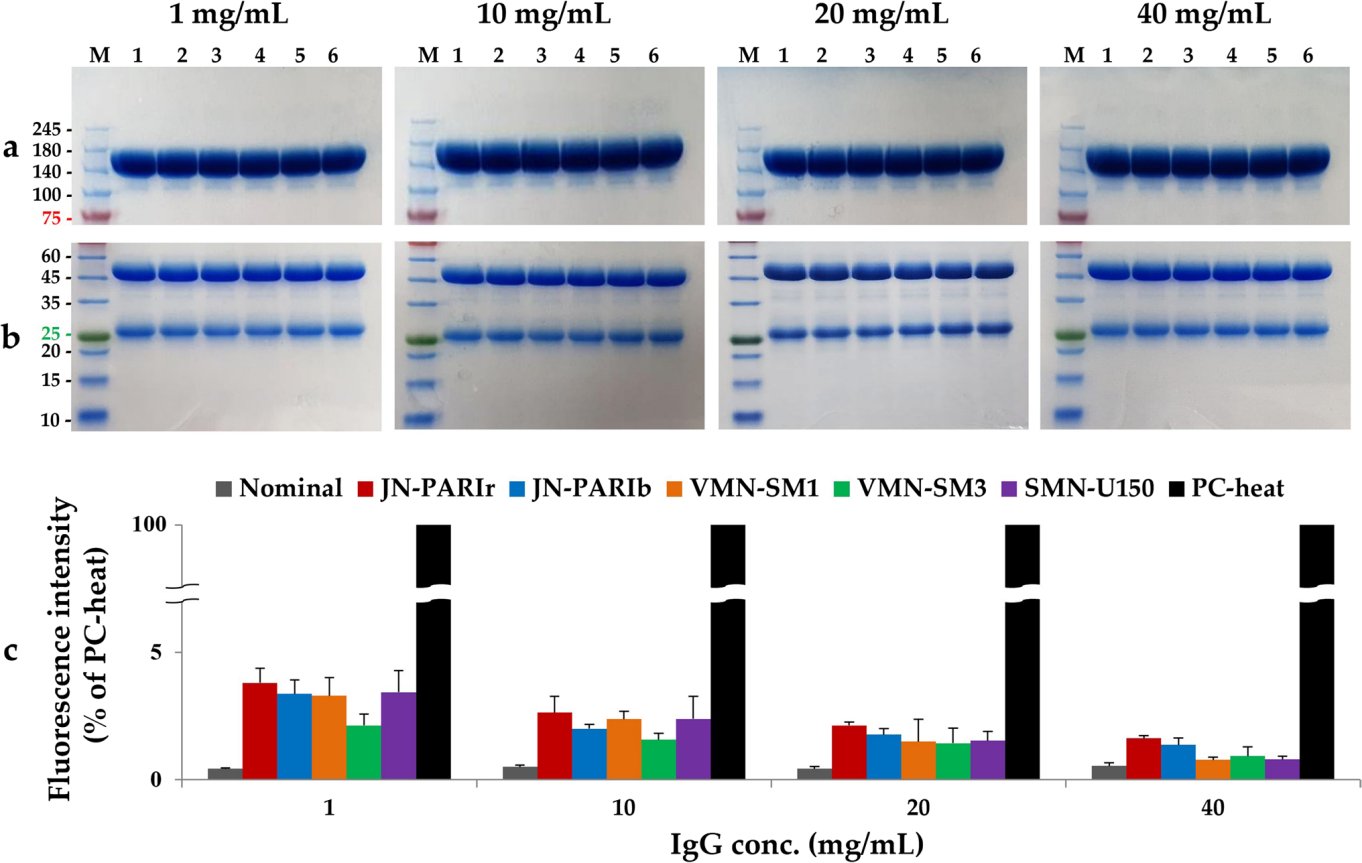
Representative native PAGE (**a**) and SDS-PAGE (**b**) analyses of IgG aerosols generated by the five nebulizers; molecular weight standards (lane M), non-nebulized nominal (lane 1), JN-PARIr (lane 2), JN-PARIb (lane 3), VMN-SM1 (lane 4), VMN-SM3 (lane 5), and SMN-U150 (lane 6). Aggregation analysis (**c**) for 100 μg of IgG aerosols emitted; non-nebulized nominal IgG: grey bars, JN-PARIr: red bars, JN-PARIb: blue bars, VMN-SM1: orange bars, VMN-SM3: green bars, SMN-U150: purple bars, and PC-heat: black bars. The aggregation of non-nebulized nominal IgG–heat (PC-heat) was defined as 100%, and the relative aggregation of IgG was calculated

As shown in Fig. 7a and b, all IgG aerosol samples showed common antibody characteristics in both reducing and non-reducing gels, as in previous studies [32, 33]. The non-nebulized nominal and nebulized IgG showed the same band patterns on the gels in all samples of different types of nebulizers, suggesting that IgG remained intact after nebulization. In the non-reducing condition, one major band with a molecular weight of 150 kDa was observed, whereas two bands of 50 kDa (heavy chains) and 25 kDa (light chains) were observed in the reducing condition Fig. 7a and b. Aggregates and fragments of IgG were not observed in IgG aerosols based on electrophoretic tests. However, the results of the aggregation analysis using a fluorescent dye Fig. 7c confirmed that aggregates were detected in all IgG aerosol samples because the fluorescence intensity was higher in the nebulized IgG than in the non-nebulized nominal samples. The increased fluorescence intensity corresponds to approximately 1–4% of the total 100 μg of IgG used in the aggregation analysis. No significant difference in fluorescence intensity was observed according to the nebulizer at IgG at the same concentration. These results show that IgG aggregates generated after aerosolization by the nebulizer can be detected by an aggregation assay using a fluorescent dye, but they are difficult to detect by the traditional gel electrophoresis method.

### Delivery Dose of IgG in Mice

**Fig. 8:**
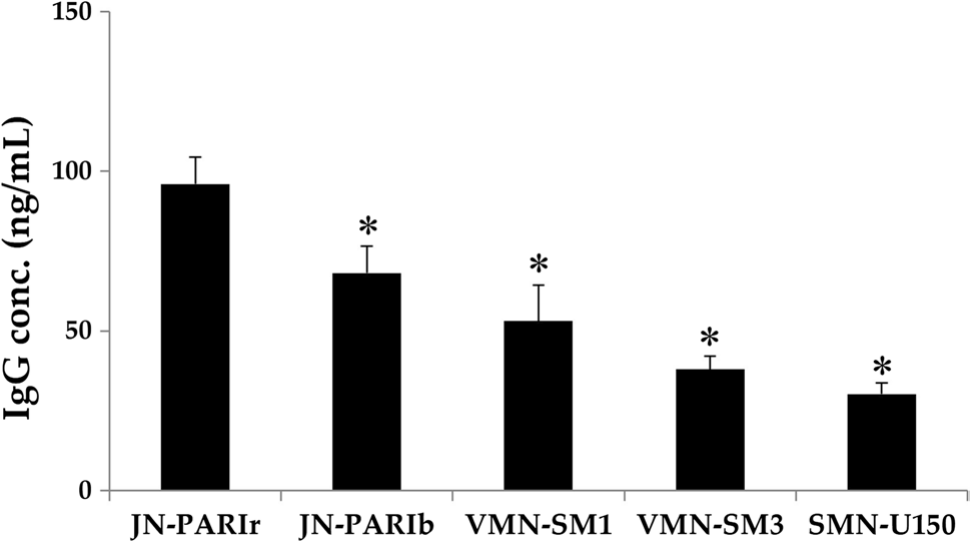
Levels of IgG in bronchoalveolar lavage fluid (BALF) from mice after exposure to 2 mg IgG (1 mg/mL) using the five different nebulizers. All data are presented as mean ± SD value. Asterisk (*) *p* < 0.05 *versus* JN-PARIr

To compare the amount of IgG delivered by five nebulizers, mice were exposed to 2 mg IgG (1 mg/mL). Large proteins can be detected in BALF because they cannot be rapidly absorbed from the lungs into the blood due to the limited permeability of the alveolar epithelium and capillary endothelium [34]. VMN-SM3 and SMN-U150, which had high output rate, short nebulization time, and large aerosol droplet size, had the lowest IgG concentrations in BALF at 38 ng/mL and 30 ng/mL, respectively Fig. 8. On the other hand, the JN-PARIr nebulizer achieved significantly higher IgG delivery in BALF at a level of 95 ng/mL than those of other nebulizers. This is because JN-PARIr has the smallest aerosol droplet sizes and the longest nebulization time, although it has a high residual volume. Interestingly, JN-PARIb and VMN-SM1 with similar aerosol droplet sizes and same nebulization time had different output rates of 0.18 mL/min and 0.31 mL/min, respectively, but no significant difference was observed at IgG level of BALF. These results support that the output rate is not the only performance indicator for the nebulizer drug delivery efficiency.

## Discussion

Since small molecule drugs commonly used in nebulizers are stored at RT and used directly, attention was not paid to the effect of drug temperature on nebulization performance. Therapeutic antibody products are stored and transported under refrigerated conditions to ensure that their quality remains consistent. In this study, the nebulization performance of the nebulizer according to the temperature and the concentration of IgG solution, the stability of aerosolized antibodies, and the amount delivered in the lungs were evaluated for different types of nebulizers.

The performance of the jet nebulizers did not differ depending on low temperature or the concentration of IgG solution, whereas the mesh nebulizers exhibited differences in nebulization times and output rates. Mitta *et al.* showed that the output rate of a jet nebulizer under different solution temperatures differed only up to 1 min, after which there was no difference [35]. This phenomenon can be described as a result of temperature changes in the reservoir during the nebulization with jet nebulizers. Kongerud *et al.* showed that the output of the jet nebulizer increases with increasing room temperature. In order to minimize the influence of the room temperature change, the room temperature was fixed at 20–22°C [36].

All of the mesh nebulizers were affected by low temperature and the concentration of IgG solution, which reduced the output rate and increased the nebulization time. The PZT in mesh nebulizer usually vibrates the most at the resonance frequency with the minimum impedance and has the highest output rate [37, 38]. Figure 4b shows that low temperature and viscosity of IgG solution can affect the output rate of the mesh nebulizer by increasing the impedance of the piezoelectric vibrating element. We demonstrated the relationship between the output rate of the nebulizer and the liquid temperature in the mesh nebulizer. The impedance of the piezoelectric vibrating element on Cold_S was higher than that of RTequ_IgG Fig. 4b. However, the increase in impedance due to low temperatures does not appear to be maintained continuously during nebulization. Because of the ambient temperature and the heat generated by the high energy input of PZT during nebulization, the output rate gradually recovered as the temperature of Cold_S increased Fig. 1 [39].

Since the viscosity of the IgG solution was measured at 25 degrees, there is a limitation that measurement values cannot be provided under low temperature conditions Fig. 4a. But, it has been reported that the viscosity of solution increases as the temperature decreases according to viscosity–temperature correlation [40, 41]. Therefore, the actual viscosity of cold IgG is expected to be higher than that of IgG measured at 25°C. In IgG nebulization results, the mesh nebulizer with a smaller output rate was found to be more effective at reducing the output rate by a low temperature and viscous solution. Therefore, direct use of refrigerated high-viscosity therapeutic proteins or antibodies in mesh nebulizers with low output rates can cause excessive fluctuations in drug delivery efficiency.

The jet nebulizers rapidly reduced IgG temperature in the reservoir to below RT during nebulization and increased IgG concentration in the residual volume Figs. 5 and 6. Cockcroft *et al.* also showed that the solute concentration remaining in the jet nebulizer increased and the temperature of the solution decreased from 24.5 to 13.5°C within 2 min after the start of nebulization [42]. They explained that evaporative water losses from jet nebulizers produce temperature drop, and increasing concentration of solute remaining in the nebulizer.

IgG aerosols generated by the mesh nebulizers can maintain biological activity due to IgG has a high melting point of 65°C, but there may still be potential for protein aggregation to be triggered and an immunogenic response [43]. However, temperature rise with nebulization time should be considered, especially when nebulization heat-sensitive proteins with mesh nebulizer.

Over the past several decades, small-molecule drugs approved for inhalation therapies are relatively free of drug stability issues, but therapeutic proteins can induce immunogenicity due to aggregate which was generated by temperature changes and shear forces during nebulization [44, 45]. It has been reported that mesh nebulizer had lower shear forces during droplets’ production in comparison with jet nebulizer [46]. The absence of IgG aggregates and fragments based on gel electrophoresis using two different gel conditions was observed Fig. 7a and b [47]. However, in the aggregation assay using a fluorescent dye, it was confirmed that 1–4% of the total amount of 100 μg of IgG became aggregated in all nebulizers Fig. 7c. This result will depend on the sample size and sensitivity used in the analysis methods for quantification of aggregates.

We observed foaming in the reservoir while nebulization IgG with a mesh nebulizer. In the previous study, 1 mg/mL dornase alfa (Pulmozyme®, recombinant human deoxyribonuclease) showed the same nebulization performance as saline without residual volume and foam [25]. The performance of mesh nebulizer is dependent on the physicochemical properties of the therapeutic protein, which can affect the efficiency of antibody delivery to the lungs. Thus, antibodies for inhalation therapy may require suitable excipients and antifoaming agents that stabilize the molecular entity against air-liquid interface generation.

The mesh nebulizers are generally known to be more effective in drug delivery efficiency than jet nebulizer because of their high output rate and small residual volume [8, 16]. Among the mesh nebulizers, IgG concentration in the BALF of mice was the lowest for VMN-SM3 and SMN-U150, which had a higher output rate and a shorter nebulization time than VMN-SM1 Fig. 8. While the JN-PARIb and VMN-SM1 nebulizers had the same exposure time of 7 min, their aerosol droplet size and output rate were differed. Specifically, the JN-PARIb had a droplet size of 5.55 μm and an output rate of 0.18 mL/min, whereas the VMN-SM1 had a droplet size of 4.72 μm and an output rate of 0.25 mL/min. The IgG concentrations in BALF for JN-PARIb and VMN-SM1 were not significantly different, measuring at 57.5 and 42.0 ng/mL, respectively*.* As only part of the aerosols emitted by the nebulizer are delivered to the lungs during the inspiration phase of the respiratory cycle, the higher the output rate of the nebulizer, the more drug is wasted during the expiration phase. The aerosol droplet size is an important parameter for drug delivery efficiency [48, 49], and the output rate also should be considered to reduce drug wastage and increase the drug delivery efficiency. These results are limited due to the use of anesthetized mice and not using nebulizers with the same aerosol droplet size in this study.

## Conclusion

Our results clearly showed that the output rate of the mesh nebulizers decreased at low temperature and high concentration of IgG solution, whereas the jet nebulizer was unaffected. This phenomenon is because the low temperature and the viscosity of IgG solution increase the impedance and change the resonance frequency of the PZT thereby affecting the output rate of the mesh nebulizer. We also found that a simple process of temperature equilibrating the cold IgG solution at RT before nebulization can solve the problem of mesh nebulizer’s output rate reduced by low temperature of solution. It was confirmed that about 1–4% aggregates were generated in a total 100 μg IgG through aggregation analysis using a fluorescent dye in all nebulizers. The output rate of the nebulizer alone does not seem to correlate with the drug delivery efficiency because the higher the output rate, the lower the concentration of IgG in BALF. For better efficient delivery of the therapeutic antibody, the respiratory synchronized output rate, temperature in use, and physicochemical properties of the therapeutic antibody should be considered.
